# Epidemiological and economic consequences of purchasing livestock infected with *Mycobacterium avium* subsp. *paratuberculosis*

**DOI:** 10.1186/s12917-017-1119-z

**Published:** 2017-06-27

**Authors:** Carsten Kirkeby, Kaare Græsbøll, Søren Saxmose Nielsen, Nils Toft, Tariq Halasa

**Affiliations:** 1 National Veterinary Institute, Technical University of Denmark, Kemitorvet, bygning 204, 2800 Kgs. Lyngby, Denmark; 2 DTU Compute, Section for Dynamical Systems, Department of Applied Mathematics and Computer Science, Technical University of Denmark, Richard Petersens Plads, Bygning 324, 2800 Kgs. Lyngby, Denmark; 3 Department of Large Animal Sciences, Section for Animal Welfare and DiseaseControl, University of Copenhagen, Grønnegaardsvej 8, 1870 Frb. C, København, Denmark

## Abstract

**Background:**

Paratuberculosis (PTB) is a chronic disease which may lead to reduced milk yield, lower animal welfare and death in cattle. The causative agent is *Mycobacterium avium* subsp. *paratuberculosis* (MAP). The economic consequences are particularly important incentives in the control and eradication of the infection.

One strategy to control PTB in a herd is to purchase animals from farms with a low risk of MAP infection. We wanted to investigate the epidemiological and economic consequences of buying livestock from different supplier farms of low, medium or high risk, as well as farms with unknown status. We also wanted to estimate the probability of spontaneous fadeout if the farmer of an initially MAP-free herd bought a specified number of infected animals in a single year, or continually bought infected animals. This was achieved through simulation modeling, and the effects of consistently introducing one, five or ten infected animals annually into an initially infection-free herd was also modeled.

**Results:**

Our findings show that once infected, a farm can relatively safely purchase animals from other low and medium-risk farms without experiencing an increase in the prevalence, highlighting the importance of certification programmes. Furthermore, farms free of MAP are highly susceptible and cannot purchase more than a small number of animals per year without having a high risk of being infected. The probability of spontaneous fadeout after 10 years was 82% when introducing a single infected animal into an initially MAP-free herd. When purchasing ten infected animals, this probability was 46%. The continual purchase of infected animals resulted in very low probabilities of spontaneous fadeout.

**Conclusions:**

We demonstrated that MAP-free farms can purchase a small number of animals, preferably from certified farms, each year and still remain free of MAP. Already infected farms have little risk of increasing the prevalence on a farm when purchasing animals from other farms.

## Background

Paratuberculosis (PTB) is a chronic infection caused by *Mycobacterium avium* ssp. *paratuberculosis* (MAP), occurring in dairy cattle. Infections can be latent for many years [1], but infected cows can shed large amounts of MAP [2]. After the latent state, animals can become clinical with fatal diarrhea. Symptoms in infected cows include reduced milk yield and weight loss [3, 4], the economic implications of which may motivate farmers to reduce the prevalence. Furthermore, reduced animal health and welfare, and the potential benefits of being able to certify a herd free of infection can also serve as motivators for farmers to lower the prevalence [5].

Around half of the Danish dairy herds are open herds, and the farmers purchase cattle (often pregnant heifers) either on a regular or irregular basis. This is similar to other countries like the USA, with 34% closed herds [6] and 47% in the Netherlands [7]. Farmers purchase cattle for many reasons, e.g. to improve the genetic potential of the herd, to increase the herd size or to restore the herd size following a disease outbreak. Approximately half of the Danish open herds use a single supplier. Using fewer suppliers may potentially reduce the risk of infection if the chosen supplier has a low prevalence. In contrast, the risk of purchasing PTB-infected cattle can be diluted by using more than one supplier, if the prevalence is low among suppliers. A certification system, as introduced in some countries, can help the farmer to reduce the risk of buying infected animals [5, 8–12].

There are approximately 3000 Danish dairy herds, with an average herd size of about 200 cows. The number of herds has reduced from approximately 12,000 in the past 20 years, while the number of cows has remained relatively stable, thus greatly increasing the herd-size and the need for purchase of livestock. Denmark has a voluntary control programme for MAP where participating herds are required to test all lactating cows four times per year, or use a risk-based test strategy where cows are tested one time per year unless they have a positive test [13]. Testing is done using a milk ELISA (IDvet, Graebels, France), and a number of recommendations are associated to the test-positive animals [14]. About 25% of the Danish farms are enrolled in the MAP control programme. Cows that have two positive tests are regarded as high-risk animals and hence farmers are recommended to cull these animals. Cows with variable results are still considered potentially infectious, but farmers are not recommended to cull these cows. The repeated testing increases the global test sensitivity [14]. Furthermore, herds participating in the control programme for MAP are advised on how to optimize the hygiene on the farm in order to break transmission routes, e.g. cleaning of calving areas and avoiding feeding calves with colostrum and waste milk from infected cows [15]. The between-herd prevalence of MAP has been estimated to be >80%, and it has been estimated that more than 75% of the herds enrolled in the MAP control programme were infected [14]. The within-herd prevalence of MAP infection in Danish dairy herds has also been investigated for herds that were not enrolled in the control programme: The median within-herd prevalence was estimated to be 5.6% of cows in the herds, but the prevalence in one herd was 45% [15]. Danish dairy cattle farms can be certified with a given MAP risk score [5, 8].

Many sellers have a low probability of infection in the cattle, and it might be considered safe to purchase from a farm with unknown risk. Where farmers use multiple suppliers and only occasionally purchase an infected animal, the probability of infection might be negligible. It is important to quantify the epidemiological and economic impact of cattle purchases on the risk of MAP introduction and spread within a dairy cattle herd. This is influenced by the MAP prevalence within the buying and selling herds, the number and frequency of cattle purchases and the number of suppliers. Knowledge of these risks would enable the provision of recommendations to help limit MAP introduction and/or spread within dairy cattle herds. Furthermore, MAP-free herds needing to purchase animals can be assisted in maintaining their free status. Therefore, our first aim was to assess the impact of purchasing cattle from herds with low, medium or high risk, or from farms with unknown status (random risk). We wanted to investigate these scenarios for both a MAP-free herd and a herd with median prevalence.

Secondly, we wanted to estimate the probability of infection fadeout after 10 years in an open, infection-free herd, where one or more infected animals were introduced at the beginning of the simulations, and to estimate the probability of acquiring a persistent infection in these circumstances.

In this study, we investigated the impact of purchase strategies by simulating combinations of situations for a dairy farm, namely: purchase from single vs. multiple suppliers; purchase of one, five or ten animals per year, and purchase from random farms (applying no MAP control measures) or farms with low, medium or high probability of infection. We also simulated an infection-free herd where the farmer bought one, five or ten animals either yearly, in a single year, or continually over 10 years. We then evaluated the resulting prevalence and probability of fadeout for all simulations.

## Methods

We used the iCull PTB simulation model, which is a bio-economic model that simulates a dairy farm using single-day time steps [15]. The model simulates a farm with 200 dairy cows, corresponding to a medium-sized Danish dairy herd. The model is a mechanistic, stochastic simulation model that simulated the individual cows in great detail, e.g. with individual lactation curves and SCC curves based on data. The iCull PTB model runs in daily time steps. Animals enter the herd as calves, are reared as heifers and later milked as cows. The farmer takes weekly decisions on which cows to cull if there are more than 200 cows present in the herd, thus keeping the number of cows stable [15]. In this study, we simulated that the farmer purchased one, five or ten pregnant heifers per year. We simulated both a situation where the farmer had a single supplier of animals, and a situation where the farmer bought from multiple suppliers (see description below). Within the model, cows feed, lactate and are inseminated and dried off as in a real herd. In the model, two thirds of culled animals are culled involuntarily due to diseases (such as lameness and mastitis) or acute injuries. This means that the farmer can make decisions for one third of the culled animals, thus resembling a real farm.

The model simulated spread of MAP between animals through the environment: bacteria are shed by infected animals every day. Accumulated bacteria followed a survival curve estimated from data. The probability of infection from the environment is based on the amount of bacteria present in the farm section where each animal is located. Additionally, animals can be infected in utero, through colostrum and waste milk [15]. If no new animals are infected from the environment or other transmission routes, disease fadeout occurs. We here defined fadeout as a permanent situation where no new animals were infected during the simulations. However, MAP bacteria can still be present in the environment for some time without infecting new animals.

The purchases were evenly spread over the year. We simulated purchases of pregnant heifers from other farms, which is a common practice in Denmark and other countries such as France [16]. Purchased pregnant heifers were introduced into the heifer section of the housing. The number of days remaining before calving was randomly drawn from a uniform distribution between 42 and 280 days after insemination. In this study, we assume that the risk of infection from an animal bought from a supplier herd is the same as the prevalence in the supplier herd, resembling that the pregnant heifer came from another farm. In Denmark, it is common for farmers to trade animals directly or through a cattle market. The probability of infection in the purchased animals was modeled according to each scenario (see below). All other properties of the purchased heifers were generated from the same distributions used in the model by [15]. In this study, we simulated for 10 years and repeated the stochastic simulations 500 times, which was previously found to be appropriate for convergence [15]. In this study, no control actions against the spread of MAP were simulated.

### Open herd scenarios

We simulated a herd with a stable herd size of 200 cows and a steady prevalence of either 0% or 5.6%. A 10-year period was chosen for this study, though it should be noted that an increase in infection prevalence or an introduction of MAP would have consequences spanning more than 10 years if no control or eradication measures were implemented. All purchased animals were pregnant heifers, as this is common practice in Denmark.

The model simulates a reduced milk production in the subclinical phase [15]. If there are infected animals in the herd, they are culled when they are detected, preventing the farmer from automatically culling the lowest producing animals. Thus the model captures that low producing cows are kept in the herd for longer time than normal. The economic calculations did not include salary for personnel or expenses such as machines and housing. Neither did we include changes in the feed conversion ratio of infected animals or potential consequences for trade. The model simulates other expenses like insemination (16.1 EUR), feed (0.133 EUR per feed unit) and the destruction of dead animals (64.8 EUR) [15]. The price of a pregnant heifer was set to EUR 1275 [17]. Each milk-ELISA cost 5.3 EUR [15].

In the simulations, the farmer could buy cattle from random herds with unknown probability of infection, or from herds certified with a low, medium or high probability of infection. We used the true within-herd prevalence in Danish herds based on [15] as the probability that a purchased animal from a given farm was infected. Therefore, in the scenarios where random suppliers were used, the probability of infection in the purchased animals was sampled from the empirical distribution of true prevalence found in 102 tested Danish herds. We also simulated three risk levels when purchasing animals: low risk, medium risk and high risk. Low risk animals had 0% to 5% probability of being infected at purchase. Medium risk animals had 5% to 15% probability of being infected, and high risk animals had 15% to 45% probability of being infected at purchase. These intervals were chosen based on the empirical distribution of within-herd prevalence in Denmark [15]. For each purchase, the probability of infection from the herd of origin was drawn from a uniform distribution between the numbers given for the risk levels described above. Whether the purchased animal was infected or not was then decided using a binomial distribution based on this probability. This resembles that a farmer purchase from a farm with a given risk level where the animal has a probability of being infected. We simulated both scenarios where the farmer purchased from a single supplier and from multiple suppliers. If multiple suppliers were used, the probability of infection in each purchased cow was redrawn from the respective distribution (described above) for every purchase.

**Fig. 1:**
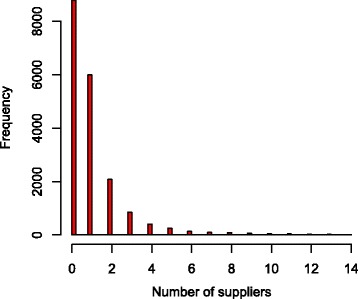
The frequency distribution of the number of suppliers used per farm. This figure is based on a dataset with the number of suppliers for 19,015 Danish dairy herds registered between 01 March 2014 and 28 February 2015, using fewer than 50 suppliers. In this plot, only farms using fewer than 15 suppliers are shown

The number of cattle purchased annually is likely to have an impact on the prevalence and thus on the economic output. We therefore simulated scenarios where farmers purchased one, five or ten animals per year. We also evaluated the impact of using single or multiple suppliers. For this purpose, we used a dataset with the number of suppliers for 19,056 Danish dairy herds registered between 01 March 2014 and 28 February 2015. Of these herds, 19,015 used fewer than 50 suppliers in that year (Fig. 1). In the scenario where multiple suppliers were used, we sampled from the empirical distribution of the number of suppliers for each farm. However, we limited the number to a maximum of 50 suppliers since we presumed that farms with more than 50 suppliers were not dairy farms. In practice, however, the maximum number of possible suppliers was ten because in this study the farmer bought either one, five or ten animals per year.

We simulated all combinations of scenarios with random, low, medium or high-risk purchases of one, five or ten heifers per year from single and multiple suppliers. All simulations included a burn-in period of 3 years and thereafter we simulated for 10 years.

### Infection fadeout

Infection fadeout is when the farm becomes free of MAP infections. It is important to estimate the probability of infection fadeout in order to make informed decisions on factors such as the implementation of disease control actions. In order to estimate the probability of infection fadeout in a herd, we simulated an initially disease-free herd under different scenarios where MAP infection was introduced. We first wanted to estimate the probability of infection fadeout in a farm without MAP, where the farmer bought one to ten infected animals in the first year only. These simulations were run for 10 years in order to estimate the probability of infection fadeout over time. The probability of infection fadeout was calculated from 500 simulations.

We then wanted to estimate the probability of disease fadeout when the farmer of an initially MAP-free herd consistently bought one to ten animals per year. This was achieved by running simulations where the farmer bought a fixed number of animals per year with a 100% probability of infection. We then calculated what percentage of the 500 simulations resulted in a fadeout of disease after 1 to 10 simulated years.

### Model updates

We used the iCull PTB model version 9.1 in this study [15]. This model included monthly milk testing in order to observe the milk yield for each cow, which was updated from an earlier version that used quarterly testing. The observed milk production is affected by the infection status [18] and as a result, the farmer would be more likely to cull infected cows with a lower milk yield. Therefore, we readjusted the force of infection and the infection probability from colostrum and waste milk in the model (using the procedure as described in [15]), in order to obtain a steady prevalence in the herd. Therefore, a fundamental presumption is that the prevalence in the simulated herd is steady without any control actions.

We also updated the model with a standard lactation fitted to every cow so that the farmer could compare the observed milk yield with the expected milk yield. This allowed for a better estimate of the milk yield level, thus helping the farmer to prioritize cows with lower production for culling.

## Results

The number of suppliers for each farm is shown in Fig. 1. Of the 19,056 farms, 51% had no suppliers, meaning that they were closed herds, and 49% had one or more suppliers.

### Open herd scenarios

**Fig. 2:**
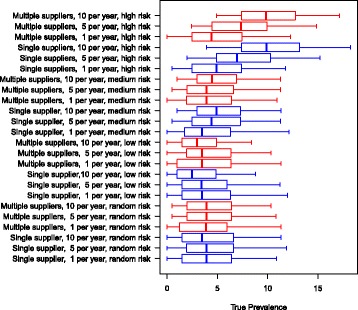
Boxplot of the resulting herd prevalence from the different scenarios after 10 simulated years for a herd with an initial MAP prevalence of 5.6%. The use of single or multiple suppliers for pregnant heifers are shown. The numbers 1, 5 and 10 represent the number of heifers purchased per year. The risk of introducing infection is given by: random risk = random supplier from the empirical distribution of herd prevalence in Denmark; low risk = 0% to 5%; medium risk = 5% to 15%, and high risk = 15% to 45%. The whiskers show the 90% simulation envelope

In the scenarios with an initial prevalence of 5.6% and where the farmer purchased animals from a low-risk farm, the herd prevalence reached 2% to 4% after 10 simulated years. When farmers purchased animals from a random farm, it resulted in 3% to 4% prevalence (Fig. 2). In these two scenarios, there was no pronounced difference between using single or multiple suppliers. In general, buying ten animals per year marginally reduced the prevalence in these two scenarios, when compared to buying one or five animals. When purchasing animals from medium and high-risk farms, the prevalence increased markedly with an increasing number of heifers bought per year, resulting in the median herd prevalence of 5% and 10%, respectively.

**Fig. 3:**
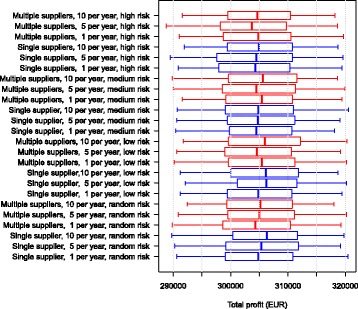
Boxplot of the net revenue in the different scenarios over 10 simulated years for a herd with an initial prevalence of 5.6%. The use of single or multiple suppliers for pregnant heifers are shown. The numbers 1, 5 and 10 represent the number of heifers purchased per year. The risk of introducing infection is given by: random risk = random supplier from the empirical distribution of herd prevalence in Denmark; low risk = 0% to 5%; medium risk = 5% to 15%, and high risk = 15% to 45%. The whiskers show the 90% simulation envelope

The economic output in the scenarios with an initial prevalence of 5.6% ended up fairly similar in all simulations (Fig. 3).

**Fig. 4:**
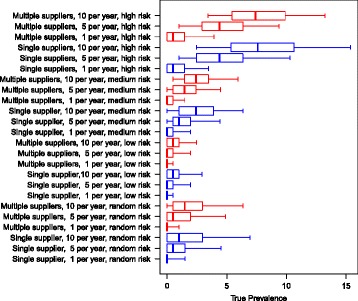
Boxplot of the resulting herd prevalence from the different scenarios after 10 simulated years for a herd with an initial prevalence of 0%. The use of single or multiple suppliers for pregnant heifers are shown The numbers 1, 5 and 10 represent the number of heifers purchased per year. The risk of introducing infection is given by: random risk = random supplier from the empirical distribution of herd prevalence in Denmark; low risk = 0% to 5%; medium risk = 5% to 15%, and high risk = 15% to 45%. The whiskers show the 90% simulation envelope

In all the scenarios with initially MAP-free herds, the resulting prevalences are clearly affected by the number of heifers purchased annually (Fig. 4). In the scenarios where the farmer purchased animals with a random risk, the resulting median prevalence was between 1% and 2%. However, if the farmer purchased only one animal from a random-risk farm per year, the median was zero and the upper 90% simulation envelope reached approximately 2%. Furthermore, 79% of the simulations resulted in a prevalence of zero (data not shown). Likewise, if the farmer bought one animal per year from a low-risk farm, 93.4% of the simulations resulted in a prevalence of zero (data not shown).

Using low-risk suppliers resulted in lower herd prevalence than the random-risk scenarios, as was expected (Fig. 4). Purchasing ten low-risk animals per year resulted in a herd prevalence of up to 3% (90% simulation envelope).

Purchasing animals from medium-risk farms resulted in a median herd prevalence of zero when purchasing one animal per year, and a median herd prevalence of 2% when purchasing ten animals per year. When purchasing animals from high-risk suppliers, the resulting median prevalence ranged between 1% (when buying one animal per year) and 8% (when buying ten animals per year). Buying ten high-risk animals per year caused the upper 90% simulation envelope to increase to 15%.

**Fig. 5:**
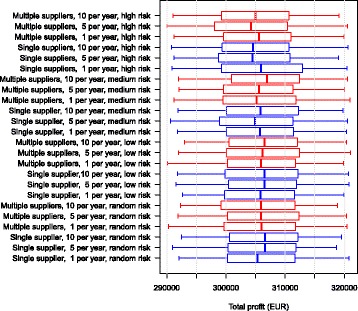
Boxplot of the net revenue in the different scenarios over 10 simulated years for a herd that was initially free of MAP. The use of single or multiple suppliers for pregnant heifers are shown. The numbers 1, 5 and 10 represent the number of heifers purchased per year. The risk of introducing infection is given by: random risk = random supplier from the empirical distribution of herd prevalence in Denmark; low risk = 0% to 5%; medium risk = 5% to 15%, and high risk = 15% to 45%. The whiskers show the 90% simulation envelope

The economic output in the scenarios with an initial freedom from MAP was also found to be very similar in all simulations (Fig. 5).

### Infection fadeout

In the simulations where the farmer purchased a number of infected animals in the first year, the probability of a fadeout decreased with the number of infected animals purchased (Table 1). For instance, when the farmer bought one infected animal, the probability of infection fadeout was 58% in the first year and 82% after 10 years. If the farmer bought ten infected animals in the first year, the probability of infection fadeout was only 16% in the first year and 46% after 10 years.

**Table 1:** The consequence of a single purchase of infected animals

	Number of animals purchased in the first year									
Simulated years	1	2	3	4	5	6	7	8	9	10
1	58	48	41	36	32	27	22	16	17	16
2	68	58	51	47	39	34	29	24	24	21
3	72	62	57	54	44	40	35	27	27	24
4	74	66	59	54	51	42	38	31	31	27
5	78	69	64	57	54	47	43	36	36	32
6	79	70	68	59	57	48	46	38	39	36
7	80	73	70	65	61	51	48	41	41	38
8	80	74	70	67	63	54	51	44	44	42
9	82	76	72	67	64	55	55	46	47	45
10	82	77	73	69	66	56	56	49	49	46

The probability (%) of fadeout after 1 to 10 simulated years when the farmer buys one to ten infected animals in the first year. For instance, in the first year, the probability of fadeout is lower when 10 animals are purchased, than when only one animal is purchased. The probabilities are calculated from 500 simulations

In the scenario where the farmer continually purchased infected animals, the probability of infection fadeout also decreased with an increasing number of infected animals purchased (Table 2). If the farmer bought one infected animal per year, there was a 99.6% probability of infection fadeout in the first year. This high percentage is influenced by the fact that the purchased heifers have not yet given birth so have not been tested, and are therefore not included in the true prevalence. As a result, the prevalence will be low before they start milking. If the farmer bought six or more animals every year, the probability of fadeout was zero in all simulated years (Table 2).

**Table 2:** The consequence of a multiple purchases of infected animals

			Number of animals purchased per year			
Simulated years	1	2	3	4	5	6
1	99.6	35.2	2.8	3.2	1.4	0.0
2	15.8	2.6	0.0	0.0	0.0	0.0
3	7.0	0.4	0.0	0.0	0.0	0.0
4	3.2	0.0	0.2	0.0	0.0	0.0
5	2.2	0.0	0.0	0.0	0.0	0.0
6	0.6	0.0	0.0	0.0	0.0	0.0
7	0.4	0.0	0.0	0.0	0.0	0.0
8	0.2	0.0	0.0	0.0	0.0	0.0
9	0.2	0.0	0.0	0.0	0.0	0.0
10	0.8	0.0	0.0	0.0	0.0	0.0

The probability of disease fadeout (calculated as percentages from 500 simulations) after 1 to 10 simulated years when buying one to six animals per year. When buying more than six animals per year, the probability of infection fadeout was always zero

## Discussion

In this study, we found that purchasing animals generally increased the herd prevalence proportionally to the probability of infection of the purchased animals and the amount of animals bought per year. However, it was still possible to buy a small number of animals from low or medium-risk farms each year and maintain a low prevalence. Furthermore, in herds that were initially MAP free, the prevalence was still zero after 10 years (within the 90% simulation envelope) if the farmer bought one heifer with random risk per year. This highlights the advantage of a certification system for farms selling livestock, if herds can be reliably divided into risk groups [8, 9, 12].

If a farmer bought a single animal from a low-risk farm per year, the simulations suggested that 93.4% of cases did not result in an infected herd. This means that in this scenario, there is only 6.6% risk of MAP infection on the farm over 10 years, again highlighting the importance of a certification programme where the farmer can actively choose to buy animals from herds with a low risk of infection over farms with a higher risk of infection.

The economic consequences did not appear to differ to a large degree, although the variation within the simulations was fairly large. As previously described in Kirkeby et al. [15], the economic consequences of having MAP within a farm are often small, so these results were as expected. In herds with an initial median prevalence of 5.6%, the purchase of heifers from low-risk farms lowered the upper 75% and 95% percentiles of simulated prevalence. This indicates that, to a certain extent, purchasing low-risk heifers can aid disease control on a farm. In these simulations, we also found that the prevalence did not change considerably even when the farmer bought animals from medium-risk suppliers. This is probably because purchased heifers do not normally come into contact with the highly susceptible calves on the farm. The risk of vertical transmission in the model is 39% and most calves born from infected dams are therefore not infected [19].

We found no noteworthy difference in prevalence between farmers using single and multiple suppliers, when these suppliers had the same risk level. We expected that using multiple suppliers would increase the risk of purchasing animals with PTB and thus introduce MAP into the herd. However, if there is only a small risk of pathogen spread within the farm from purchased animals, then a larger number of infected animals are required to increase the prevalence on the farm.

**Fig. 6:**
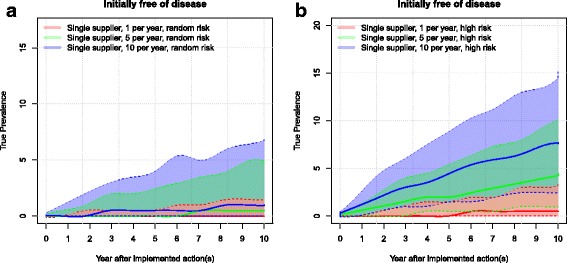
Purchase of animals with random risk of infection: the development of prevalence in a herd initially free from MAP importing animals with (**a**) random risk or purchasing animals with (**b**) high risk. The farmer can purchase 1, 5 or 10 per year. Solid lines show the median prevalence and dotted lines show the upper and lower 90% simulation envelope

**Fig. 7:**
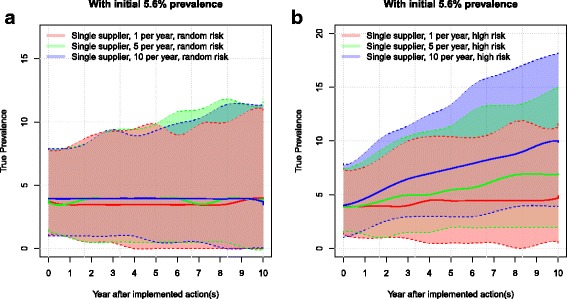
Purchase of animals with random and high risk of infection in an already infected herd: the development of prevalence in a herd with a normal level of infection purchasing animals with: (**a**) random risk and (**b**) high risk. Solid lines show the median prevalence and dotted lines show the upper and lower 90% simulation envelope

The prevalence in the simulated scenarios without fadeout (shown in Figs. 6 and 7) is steadily increasing and has not yet reached a steady state. This underlines the slowly evolving nature of PTB. We chose to look at a 10-year timespan in this study, but it is evident that the impact of disease would continue over a much longer period. The model assumes that the same management strategy is used throughout the simulated years.

A previous study [20] simulated a closed herd with endemic MAP infection, and found that the probability of infection fadeout decreased with the herd size. They used a median prevalence of 20%, which is much higher than in this model.

We found that farmers that bought one infected animal per year had a 99.6% chance of having a disease-free herd after the first year (Table 2). However, in the second year they had only a 15% chance of being MAP-free (which would also imply that the purchased and infected animal was culled). If the farmer bought two infected animals per year, he would have only a 35% chance of a MAP-free herd after the second year. The low risk of infection after the first year can be partly attributed to the fact that the true prevalence is only calculated for cows, and does not include infected (purchased) heifers or calves. Furthermore, these estimates include the purchased infected animals, so the probability of a fadeout is also affected by the probability of the purchased animals being slaughtered.

A simulation study by Pouillot et al. [21] showed that in a dairy herd with 40 cows, the maximum fadeout probability of 20% was reached after 10 years. Others [22, 23] estimated that the introduction of one infected animal into a MAP-free herd with 114 cows resulted in disease fadeout in 66% of the simulations. Marcé et al. [22] found that when one animal is purchased per year over 10 years (0.66^10^), the chance of fadeout is reduced to 2%. In the study by Lu et al. [24], it was found that an initially infection-free herd with 140 cows had a 42% probability of infection fadeout 10 years after the introduction of a single infected animal. In the present iCull model, this probability was higher at 82%. This may be the effect of a relatively low force of infection in the iCull model, which was introduced to calibrate the model to reflect the prevalence in Danish herds.

No control actions were simulated since we chose to look at the general effect of purchasing animals into the herd. Clearly, implementing control actions would increase the probability of disease fadeout, but this is beyond the scope of this study. The impact of control actions on MAP spread within Danish dairy cattle herds was recently examined by [15].

In this model, we included contamination between farm sections. This mechanism might not be relevant on an infected farm, but on an uninfected farm it could potentially transfer MAP from a newly imported heifer to the calf section. The risk of infection is higher for calves due to a decreasing susceptibility with age, and therefore in this case, contamination between farm sections would be critical [25]. The low probabilities of a disease fadeout highlight the importance of buying uninfected animals.

We here assumed that farmers bought pregnant heifers. Therefore our results are limited to this situation. Furthermore, the simulated farm is specifically built to mimic a Danish dairy herd. Consequently our results are not directly applicable to other scenarios where the farm configuration differs a lot from such a farm. The probability of fadeout in this study is dependent on the survival rate of MAP. If, for instance, another strain of MAP has a higher survival rate in the environment, it would take longer time for MAP to fadeout.

## Conclusions

To conclude, the simulations showed that it is possible for MAP-free farms to import a small number of animals each year and still remain free of MAP. If the farmer continually imports animals from certified herds with a known low risk of infection, the actual risk of infection in their herd is very low. In contrast, if the farmer buys animals from a random source, there is a substantial risk of acquiring infection in the herd.
